# Influence of the NICU on the Acoustic Isolation of a Neonatal Incubator

**DOI:** 10.3389/fped.2020.00588

**Published:** 2020-09-22

**Authors:** Virginia Puyana-Romero, Daniel Núñez-Solano, Ricardo Hernández-Molina, Edgar Jara-Muñoz

**Affiliations:** ^1^Grupo de Investigación Entornos Acústicos, Facultad de Ingeniería y Ciencias Aplicadas, Universidad de Las Américas, Quito, Ecuador; ^2^Laboratorio de Ingeniería Acústica, Universidad de Cádiz, Cádiz, Spain; ^3^Unidad de Neonatología, Hospital Metropolitano, Quito, Ecuador

**Keywords:** acoustic environment, low frequency noise, acoustic isolation, neonatal incubator, reverberation time

## Abstract

The neonatal intensive care unit (NICU) is a very noisy place as compared to the intrauterine environment. To protect the neonate's health, international guidelines suggest avoiding noise levels above 45 dB in NICUs, but this recommendation is not normally met. The incubator acoustic isolation and the acoustic features of the NICU play important roles in determining the noise measured inside the incubator. In this study, the influence of two types of rooms, one with sound-absorbent covering and the other with reverberant surfaces, on the acoustic isolation of a neonatal incubator was evaluated using three acoustic isolation indexes: the level difference, the apparent sound reduction index, and the standardized level difference. Results show that the acoustic isolation of the incubator is very poor, with a level difference below 11 dBA at all frequencies. At 62.5 Hz, the level difference measured in both rooms exhibits a negative value, indicating that the incubator amplifies the noise coming from the NICU. Isolation of the incubator is poor, and the reverberation time (RT) of the containing room influences RT of the incubator, which is consequently higher when the containing room is reverberant; for example, the incubator RT in the reverberant NICU is 0.72 s higher at 500 Hz than that in a room with sound-absorbent covering.

## Introduction

Numerous studies affirm that neonatal intensive care units (NICU) are noisy environments exceeding recommended sound levels (1–3) and that high noise levels have a significant impact on the health of preterm infants (4–8). The American Academy of Pediatrics uses the U.S. Environmental Protection Agency noise standards for hospitals (45 dB during the day and 35 dB at night) and recommends avoiding sound levels over 45 dB in NICU environments (9, 10). However, studies affirm that NICU noise levels exceed these recommendations between 70% (1) and 95.5% (2) of the time, exhibiting average noise levels ranging from 48 to 60.6 dBA (11, 12).

Although measurements indicating increases in NICU noise levels have been published over the years, recent studies show that this is still an unsolved problem. A study of different types of NICU facilities indicated an average equivalent continuous sound level (Leq) of 48 dBA inside the incubator, with the highest transient sounds at Leq = 81 dB (13); outside the incubator (also in different NICU types), two different studies reported measured noise levels of Leq = 55–65 dBA (13) and Leq = 54.7–58.1 dBA (14).

Several training programmes and procedures have been implemented to reduce NICU noise levels (15–17), but none of them has resulted in reductions to levels below those recommended. A recent observational study (18) was undertaken with the goal of reducing NICU noise levels below the recommended 45 dBA. The maximum decrement only reduced levels to 55.3 dBA even though three different procedures were applied; these included awareness and education, environmental noise reduction measurements, and unit modification procedures.

Noisy environments affect premature infants more than full-term infants, as the peripheral auditory system is not yet fully developed in premature infants (19). The womb and the impedance mismatch between air and the embryonic fluid attenuate frequencies above 500 Hz by 40 to 50 dB, and consequently, the developing fetus is not exposed to the spectrum above this frequency (20). However, the spectral content of the noisy NICU environment is completely different from that of the intrauterine environment, and the auditory system of preterm infants is not prepared for it (21). As a consequence, preterm infants show distinctive responses to environmental stress within the NICU (22). Preterm infants cannot habituate to the acoustic stimuli, even after repeated exposures (23), and their heart rate, respiratory rate, and oxygen saturation increase in noisy environments more than those of full-term infants (4, 24).

According to Vohr (25), a stay in the NICU for more than 4 days constitutes a risk factor for hearing loss in neonates. Furthermore, the noisy NICU environment causes other adverse effects on infants' health (4–8). Stressful acoustic stimuli are related to future disorders in language and attention (26, 27). Heart rate, intracranial pressure, and oxygen saturation changes are some of the stressful physiologic responses (4, 28–31) that may have a significant impact on infant neurological development (22).

The spectral content of NICUs shows a predominance of low frequencies (3, 32), with the addition of some mid-high frequency events resulting from equipment alarms and human voices (33). However, there is limited information on the specific effects caused by the low frequencies to which neonates are exposed. In that regard, a recent study suggested that if low frequency exposures have negative impacts on the health of exposed mice, which have a higher threshold for low frequencies than that for humans [375 (34) and 20 Hz, respectively], they could also have negative impacts on humans (35). Results show that the exposure of mice to low frequency noise with levels of 70 dB over 4 weeks causes permanent imbalance and a reduction of the number of calbindin-positive hair cells in the saccule and utricle (35).

Most studies have used Leq, which is the energy contained in the entire spectrum, to evaluate noise in NICUs and incubators; there have been some exceptions, including the studies carried out by Santos et al., Hernández Molina et al., and Fernández Zacarías et al. (3, 33, 36). However, this parameter does not indicate which frequencies are dominant. The acoustic attenuation caused by the incubator dome is crucial to the evaluation of noise within the incubator, but this aspect has largely gone unnoticed in most prior studies.

The motivation of this study is to provide an approach to improving the acoustic environment of NICUs and incubators to avoid potential neonatal health issues. Thus, the primary objective of this paper is to evaluate the influence of the NICU and incubator in the transmission of noise inside the incubator.

## Methods

### Physical Space and Measurement Procedures

**Figure 1:**
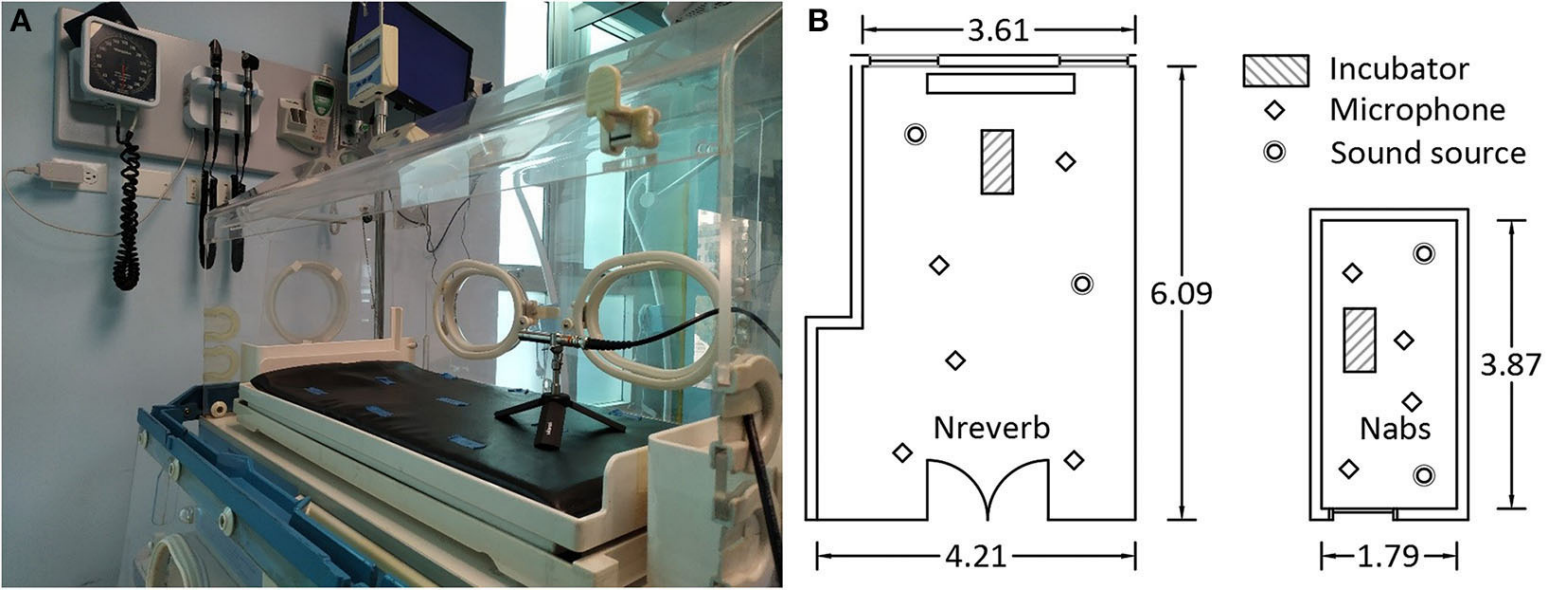
**(A)** Incubator was studied with mattress inside. **(B)** Incubator, sound source, and microphone positions in the reverberant (Nreverb) and absorbent (Nabs) rooms.

Two different rooms containing the same incubator were studied to evaluate the incubator acoustic isolation and the influence of the containing room on the noise levels. These two rooms are located at the Universidad de Las Américas (Quito, Ecuador). The first one measured 1.79 × 3.87 × 2.52 m and was in the Sound and Acoustic Engineering Department. The second one was the CS1 room in the Simulation Medic Center of the Medicine Faculty and measured 3.87 × 6.09 × 2.5 m (Figure 1B). The first room has sound-absorbent walls with an average RT of 0.15 s, while the second one is much more reverberant (RT = 1.18 s) and is similar to a NICU room. Henceforth, the first room will be referred to as the “absorbent NICU” (Nabs) and the second one as the “reverberant NICU” (Nreverb). The designation “Nabs” is used to characterize the incubator acoustically. In Nabs, nearly all of the energy is attenuated by the absorbent walls. Conversely, the Nreverb is used to study the influence of the NICU on the incubator and represents an actual reference for the characteristics of the incubators and NICUs. Acoustic insulation measurements generate very high noise levels; therefore, they must be performed in NICUs without patients. Since there was no NICU meeting this requirement, it was necessary to use another room with similar characteristics; hence, we chose Nreverb. The dimensions of the incubator are ~0.85 × 0.41 × 0.40 m. The incubator was turned off, the access portholes were closed during measurements, and the mattress was contained inside (Figure 1A).

The Simulation Medic Center staff provided the facilities and gave advise on the general features and disposition of the NICU and incubators, and the Neonatal Unit of the Santa Bárbara Clinic of Quito provided the incubator studied.

Incubator acoustic isolation was measured using the standard ISO16283-1 (37) as a reference. It was used only as a reference because this standard is suitable for rooms with volumes between 10 and 250 m^3^, and the incubator space is significantly smaller. The incubator interior was considered as the receiver room (for both groups of measurements) because of the newborns' sensitive natures and the suggestion of the ISO standard in selecting the smallest room as a receiver (to ensure that the standardized level difference (DnT) is not overestimated) (37). To ensure straightforward data interpretation and to obtain more representative outcomes, the acoustic isolation was calculated in octave bands and not in 1/3 octave bands, as the ISO16283-1 recommends.

#### Incubator Acoustic Isolation in Nabs Room (Figure 1B)

The Nabs dimensions only provided for four microphone locations, requiring the sources to be placed at the corners to measure noise levels outside of the incubator. Levels were measured twice for each microphone–source combination (i.e., sixteen mic–source measurements). For measurements of the incubator energy distribution, 15 microphone positions were used inside the incubator; this matches the procedure used for small rooms in previous studies conducted by the authors (38, 39). A total of 60 noise level measurements were conducted, with four measurements in each of 15 positions. Subsequently, reverberation time (RT) and background noise were measured in the same fifteen microphone locations for each of the two impulsive sound source (balloon burst) locations.

#### Incubator Acoustic Isolation in Nreverb Room (Figure 1B)

Noise level measurements outside of the incubator were carried out using two sound sources and five microphone positions. Levels were measured twice for each microphone–source combination (i.e., 20 mic–source measurements). The number of measurements and mic-source combinations used to evaluate noise levels within the incubator, the background noise, and the RT were the same as those used in Nabs.

The ISO16283-1 standard recommends conducting measurements at low frequencies (from 50 to 80 Hz) when one or both rooms have a volume of <25 m^3^. In such small rooms, it is not easy to achieve a diffuse field, and additional measurements are required. In our case, the receiver has a volume of V = 0.147 m^3^ < 25 m^3^, so it was necessary to conduct low frequency measurements for noise level, background noise, and RT at the receiver. Noise level and background noise must be measured at least in four corners and the RT in a position other than a corner. The measurements carried out in the 15 incubator locations fulfill the previous requirements. Four additional measurements were also made in the corners of Nabsorb since its volume is below 25 m^3^. Although it is not required by ISO16283-1, the RTs of Nabs and Nreverb were also measured.

As a result of the measurements described above, four groups of acoustic parameters were obtained in each room for each of the octave bands studied; these included noise levels inside the incubator, L_in_ (L_in___63_, L_in_125_, L_in_250_, L_in_500_, L_in_1000_, L_in_2000_, L_in_4000_), noise levels outside the incubator, L_out_ (L_out___63_, L_out_125_, L_out_250_, L_out_500_, L_out_1000_, L_out_2000_, L_out_4000_), reverberation time inside the incubator, RT_in_ (RT_in_63_, RT_in_125_, RT_in_250_, RT_in_500_, RT_in_1000_, RT_in_2000_, RT_in_4000_), and reverberation time outside the incubator, RT_out_ (RT_out63_, RT_out125_, RT_out250_, RT_out500_, RT_out1000_, RT2_2000_, RT_out 4000_).

The level difference (D), the apparent sound reduction index (R′), and DnT were calculated in this study (see ISO16283-1 for further information). The D index constitutes the simplest approach to the definition of sound insulation, and it is the level difference between the emission and receiver rooms. However, it is not suitable for comparing constructive solutions because the acoustic isolation also depends on the acoustic treatment of the receiver room. The second index is typically used to compare different materials, and it is the reference value used by manufacturers to characterize products acoustically. The third index is used for evaluating complex building solutions and for comparing *in situ* measurements, and it considers the RT measured in the receiver room. Considering that the scope of the present study does not include comparing different construction solutions because we were using the same incubator, indexes D and DnT were expected to yield more useful results.

The Mann–Whitney *U* test was applied to check the statistical significance of the differences between the measurements of the four subsets of acoustic parameters (L_in_, L_out_, RT_in_, and RT_out_) conducted in Nabs and Nreverb. The same test was applied to the RT measurements conducted in Nreverb, outside and inside the incubator (RT_in_Nreverb_ and RT_out_Nreverb_). The acoustic indexes of both rooms were calculated with each sound source considered independently and for the overall number of measurements. The Mann–Whitney *U* test was also applied to determine if the differences between the acoustic indexes obtained for each room are statistically significant. Pearson's correlation coefficient was also calculated to determine if there is a relationship between the RT and the noise indicators and between RT_in_ and RT_out_.

### Room Impulse Response Measurements

The impulse response of a room (RIR) is a signal that contains information on the direct sound and early and late reflections arising when the room is excited by a sound source (38, 40). Additionally, the RIR describes the room sound energy and its decay; thus, by gathering the RIR of the incubator or NICU, it is possible to determine RTs and the sound pressure levels (SPLs). Subsequently, the RT and SPL may be used to estimate the acoustic isolation of the incubator.

The electroacoustic chain used for the measurements consists of a Gras 1/2" CCP free-field microphone, an external AVID sound card (i.e., AD/DA converter), and a computer-running Matlab to gather the data. In addition, the ITA toolbox (41) was used to obtain the RT and the SPL at each measurement position. For measurements of the incubator RIR, balloons were used instead of an omnidirectional source. A CESVA omnidirectional source and Gaussian white noise were used for measurements of RIR in the Nabs and Nreverb.

## Results

Since the measured noise levels were 10 dB higher than the background noise, no background noise correction was applied. Measurements of RT, designated T30, were considered in this study; T30 is a measure of the time in which the SPL decays 30 dB, multiplied by 2.

The Kolmogorov–Smirnoff test was calculated to establish the goodness of fit to normality for the variables studied. Since some variables did not satisfy normality criteria, the non-parametric Mann–Whitney *U* test was applied as an alternative to parametric statistical studies. The data used for the analyses meet the assumptions necessary for the application of this test (42). The groups of the categorical-dependent variables are Nabs and Nreverb. The independent variables are the acoustic parameters and the acoustic isolation indexes in octave bands.

Results show that there are statistically significant differences between Nreverb and Nabs for all noise levels except for L_out___1000_, L_in_1000_, L_in_2000_, and L_in_4000_, with a significance level of 5% (Table 1). Within the frequency range, 250–4,000 Hz, the noise levels inside and outside the incubator are higher in Nreverb than they are in Nabs, but are lower in Nreverb for the other frequencies. Similarly, the RTs exhibit statistically significant differences between the rooms at all band frequencies and the values in Nreverb are higher than those in Nabs, with the exception of RT_in_63_. The RTs measured at 63 and 125 Hz in Nreverb are significantly higher inside the incubator than those measured outside. Still, the reverse is true (i.e., higher RTs outside the incubator) for frequencies from 500 to 4,000 Hz. No significant differences were found at 250 Hz.

**Table 1:** Mann–Whitney test applied to the acoustic parameters measured and to the noise indexes (level difference D and standardized level difference DnT) calculated for Nreverb and Nabs.

**Noise levels**			**Reverberation times**			**Noise indexes**		
**Variable**	***p*-value**	**Subset of higher mean rank**	**Variable**	***p*-value**	**Subset of higher mean rank**	**Variable**	***p*-value**	**Subset of higher mean rank**
L_out_63_	0.000^*^	Nabs	RT_out_63_	0.000^*^	Nreverb	D_63_	0.037^*^	Nreverb
L_out_125_	0.000^*^	Nabs	RT_out_125_	0.000^*^	Nreverb	D_125_	0.037^*^	Nabs
L_out_250_	0.000^*^	Nreverb	RT_out_250_	0.000^*^	Nreverb	D_250_	0.037^*^	Nabs
L_out_500_	0.000^*^	Nreverb	RT_out_500_	0.000^*^	Nreverb	D_500_	0.037^*^	Nreverb
L_out_1000_	0.080	(Nreverb)	RT_out_1000_	0.000^*^	Nreverb	D_1000_	0.037^*^	Nreverb
L_out_2000_	0.000^*^	Nreverb	RT_out_2000_	0.000^*^	Nreverb	D_2000_	0.037^*^	Nabs
L_out_4000_	0.004^*^	Nreverb	RT_out_4000_	0.000^*^	Nreverb	D_4000_	0.037^*^	Nreverb
L_in_63_	0.000^*^	Nabs	RT_in_63_	0.000^*^	Nabs	D_nT_63_	0.050^*^	Nreverb
L_in_125_	0.000^*^	Nabs	RT_in_125_	0.000^*^	Nreverb	D_nT_125_	0.827	(Nabs)
L_in_250_	0.009^*^	Nreverb	RT_in_250_	0.000^*^	Nreverb	D_nT_250_	0.050^*^	Nreverb
L_in_500_	0.000^*^	Nreverb	RT_in_500_	0.000^*^	Nreverb	D_nT_500_	0.050^*^	Nreverb
L_in_1000_	0.174	(Nreverb)	RT_in_1000_	0.000^*^	Nreverb	D_nT_1000_	0.050^*^	Nreverb
L_in_2000_	0.692	(Nreverb)	RT_in_2000_	0.000^*^	Nreverb	D_nT_2000_	0.050^*^	Nreverb
L_in_4000_	0.224	(Nreverb)	RT_in_4000_	0.000^*^	Nreverb	D_nT_4000_	0.050^*^	Nreverb

* *p-value ≤ 0.05*.

*Subsets of mean rank in parentheses indicate that the difference between groups is not significant*.

The outcome of the Mann–Whitney *U* test applied to the isolation acoustic indexes shows that significant differences (*p*-value ≤0.05) existed between Nabs and Nreverb for all of the variables, except for D_nt_125_ and R_63_. From the data with *p*-value ≤0.05, it can be concluded that the acoustic indicators calculated for Nreverb are significantly higher than those for Nabs, with the exception of D_125_, D_250_, and D_2000_ (which are lower).

Pearson's correlation coefficient (*r*) was calculated to compare the RT outside the incubator and the acoustic isolation indexes (for all the band frequencies). The correlation coefficients with the DnT index are very high for all the band frequencies (*r* > 0.9, *p*-value <0.001). High correlations also occur with D at 63, 500, 1,000, and 4,000 Hz and with R at 125 and 1,000 Hz (*r* > 0.9, *p*-value <0.001). The same trend can be observed for the correlation coefficients resulting for the RT_in_ and R_out_ at high band frequencies, although the correlations at low frequencies are slightly lower (*r*_63_ = −0.494, *p*-value_63_ = 0.019; *r*_125_ = −0.515, *p*-value_125_ = 0.014).

**Figure 2:**
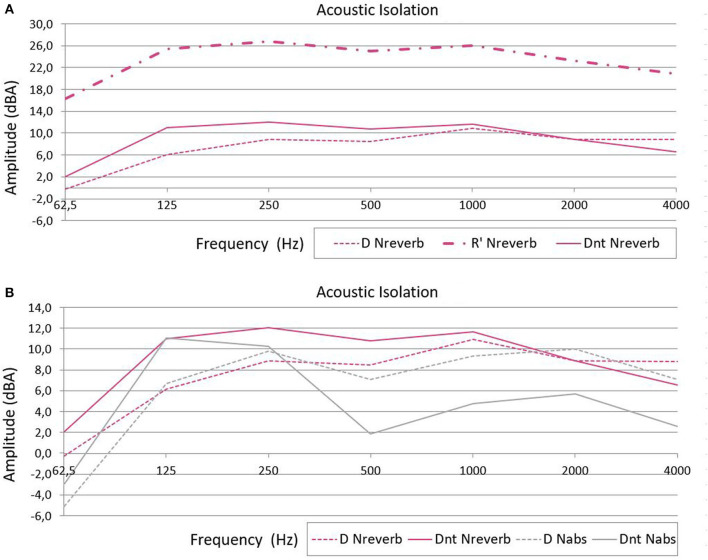
**(A)** Level difference (D), apparent sound reduction index (R′), and standardized level difference (DnT) isolation indexes (in dBA) for the incubator in the reverberant room. **(B)** Comparison of the level difference (D) and standardized level difference (DnT) isolation indexes of the acoustic absorbent (Nabs, *gray*) and reverberant (Nreverb, *pink*) rooms.

Figure 2A shows D, R′, and DnT isolation indexes for the incubator located within Nreverb. The highest D attenuation is approximately 11 dBA at 1 kHz; the poorest attenuation is at 62.5 Hz (D = −0.25 dBA), followed by 125 Hz (D = 6.15 dBA), and the attenuation for other frequencies is approximately 8.5 dBA. A comparison of DnT and D for the Nreverb room shows that DnT is higher at all frequencies except for 4,000 Hz.

Figure 2B shows D, R′, and DnT indexes for each room. The D index at 62.5 Hz is also negative in Nabs (−5.13 dBA). The DnT is higher when the incubator is in Nreverb than when the incubator is in Nabs (Figure 2B).

**Figure 3:**
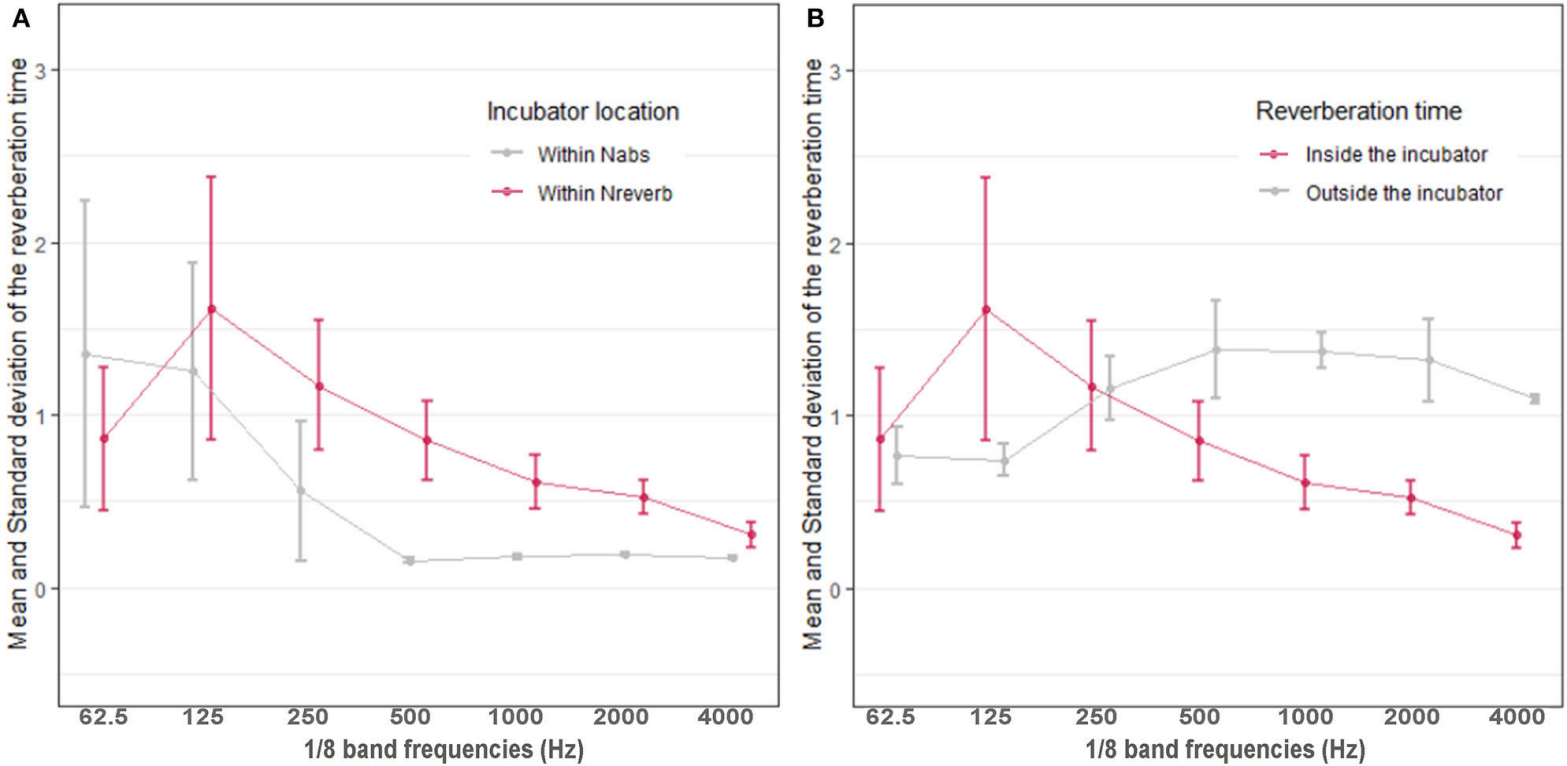
**(A)** Average reverberation time within the incubator in the reverberant (Nreverb, *pink*) and absorbent (Nabs, *gray*) rooms. **(B)** Reverberation time inside (*pink*) and outside (*gray*) the incubator in the reverberant room Nreverb.

Figure 3A shows that the average incubator RT is higher in the Nreverb than in the Nabs. However, there is an exception at 62.5 Hz; at that frequency, there was a small difference between the signal and background noise level in Nreverb, and it was not possible to measure T30. In this case, T20 was used as a substitute for T30 (T20 = time at which the SPL decays 20 dB, multiplied by 3). T30 was normally higher than T20 in the present experiments, and therefore, the T30 at 62.5 Hz could have also been higher in Nreverb than in Nabs at 62.5 Hz. Excluding that exception, the incubator RT in both rooms decreased as the frequency increased. In Nreverb, the RTs for low frequencies are higher inside the incubator than outside (Figure 3B). Furthermore, when both spaces (Nreverb and incubator) are independently excited by a 125-Hz signal, the sound persists 0.8 s longer in the incubator than it does in the NICU.

## Discussion

The statistical analysis shows that most of the acoustic parameters measured in the two rooms are different. It is important to highlight that, although the same configuration of the sound source and measuring equipment was used in both rooms, measured noise levels for frequencies from 250 to 4,000 Hz were higher in Nreverb than they were in Nabs; this illustrates that a reverberant room amplifies noise levels. The fact that noise levels at low frequencies are lower in Nreverb results from the poor isolation of the glass walls for those frequencies, which leads to a higher noise transmission to the adjoining rooms. Therefore, similar to the case for the incubator and the room in which it is located, there is an acoustic coupling of the NICUs (with a high percentage of glass) with the adjoining rooms. This is undesirable because many electronic devices located in adjoining rooms may generate low frequency noise and the glass walls cannot protect the NICU environment from this noise.

When the incubator is in Nreverb, the highest acoustic isolation D occurs at 1 kHz and is approximately 11 dBA, which means that the incubator dome only poorly attenuates the noise coming from outside. Therefore, most of the low frequency sounds coming from the heating, ventilation, and air conditioning systems (HVAC) and other incubator engines reach the neonate easily. Similarly, and with regard to mid-high frequencies, poor attenuation results with human voices and noises from equipment with alarms. The incubator isolation is so poor that the RT of the containing room influences its RT. Pearson's correlation coefficient between the RT inside and outside the incubator is higher than 0.9 (*p*-value < 0.001), supporting this statement. For example, the incubator RT at 500 Hz is 0.72 s higher in Nreverb than it is when the incubator is in Nabs.

The negative D value at 62.5 Hz in Nabs and Nreverb means that the reverberant nature of the incubator causes amplification of the noise, leading to a higher noise level inside the incubator (relative to outside) at that frequency. These findings are in accordance with the suggestions made in other reports (43, 44).

DnT is an acoustic isolation index that considers the influence of the RT of the receiver room on the isolation. It is equal to D+10^*^log(T/T_0_), where T is the measured RT at the receiver and T_0_ is the reference RT value; the ISO16283-1 recommends a T_0_ = 0.5 s. Consequently, the term 10^*^log(T/T_0_) is positive when T > 0.5 s and in that case, DnT is higher than D. Comparing DnT and D for the same Nreverb room, DnT is higher at all frequencies except for 4,000 Hz, meaning that the RT inside the incubator exceeds the reference T_0_. For example, the RT inside the incubator for a frequency of 125 Hz is 1.59 s, three times higher than the aforementioned reference T_0_ (0.5 s). In Nabs, however, the DnT equation shows that the RTs at 62.5 and 125 Hz are higher than T_0_, but they are shorter than T_0_ at the other frequencies (Figure 2B).

Although the incubator DnT is higher when the incubator is in Nreverb than it is when the incubator is in Nabs (Figure 2B), this does not mean that the acoustic isolation is higher; this is also a result of the T_0_ used in ISO16283-1. Since the incubator acoustic isolation is so poor, sound waves go in and out through the incubator walls readily. Therefore, the RT of the room in which the incubator is located affects the incubator RT (note that this phenomenon is not usually observed in the ordinary dwelling rooms for which ISO16283-1 was designed). Since the RT of Nreverb is higher than Nabs, the incubator RT is also higher when it is in Nreverb than when in Nabs, and consequently, so is DnT.

The T_0_ recommended in the ISO16382-1 standard was chosen for typical dwelling rooms containing furniture, for which the RT is reasonably independent of volume and frequency and is approximately equal to 0.5 s for most frequencies (37). According to the present results, a T_0_ = 0.5 s is not suitable for small and reverberant rooms. In this regard, the authors of (45) conducted RT measurements in furnished and unfurnished rooms; their results showed that the average RT for unfurnished rooms is ~2.5 s at frequencies below 500 Hz and decreases to 1.3 s at frequencies of 5 kHz. In addition, the standard deviations for unfurnished rooms were higher than the furnished one, which is probably because the authors evaluated rooms with different volumes (ranging from ~10 to 200 m^3^) (45). Consequently, the RTs for unfurnished rooms are volume and frequency dependent. However, it is difficult to establish a reference value for the DnT equation that suits the characteristics of the incubator, since the volume is not comparable with those studied in (45). Thus, more research on the RT reference value (T_0_) is needed to calculate the isolation indexes for small and reverberant spaces, since neither the DnT nor the D indexes show the real effect of the RT on the acoustic isolation.

Although the traditional isolation indexes used in construction seem to be inappropriate for characterizing the acoustic isolation of neonatal incubators, the present outcomes show that incubator isolation is very poor, especially at low frequencies. This is quite problematic since many studies affirm that most of the time, there are elevated low frequency intensities in NICUs (3, 32) caused by electronic devices (46). Although the information is limited, some studies suggest that low frequency exposure may have negative effects on neonates, as is the case for animals with auditory thresholds higher than those of humans (35).

There is a strong relationship between the RT outside the incubator and the DnT index for all band frequencies (*r* > 0.9, *p*-value < 0.001), although the DnT equation does not consider the RT outside, but only inside, the incubator. Moreover, the high correlation between D (L_out_-L_in_) and the RT outside the incubator implies a strong influence of the RT of the room containing the incubator on incubator isolation.

Since Nreverb is a room designed for teaching activities, it has an acoustic absorbent false ceiling not normally found in traditional NICUs. Therefore, although it is quite reverberant for its volume, in a real NICU, the reverberation times obtained would have been higher.

Not only does the incubator exhibit poor isolation, but it also amplifies sound at low frequencies; therefore, the problem of high noise levels in NICUs is worsened from the perspective of the preterm infant. It would seem that incubators must be designed for improved acoustic comfort since Coston and Aune (18) have shown that reducing NICU noise levels is complicated. Conversely, another way to decrease noise levels inside incubators is to treat NICUs acoustically, since our results show that the incubator reverberation time is influenced by the NICU reverberation time; therefore, reductions in NICU RTs will lead to reductions in the RTs and SPLs of the incubator.

## Conclusions

The results of the present study on the acoustic isolation and reverberant nature of neonatal incubators establish that incubators and traditional NICUs provide acoustic environments that are inappropriate for neonates. The results of the present study may provide clinical managers with evidentiary support for the need to redesign traditional NICUs.

## Data Availability Statement

The original contributions presented in the study are included in the article, further inquiries can be directed to the corresponding author.

## Author Contributions

VP-R conceptualized and designed the study, carried out the acoustic measurements and initial analyses, drafted the initial manuscript, and reviewed and revised the manuscript. DN-S carried out the acoustic measurements, carried out the initial analyses, drafted the initial manuscript, and reviewed and revised the manuscript. RH-M coordinated and supervised data collection and critically reviewed the manuscript and provided important intellectual content. EJ-M assessed neonatal intensive care units and electrical devices and critically reviewed the manuscript and provided important intellectual content. All authors contributed to the article and approved the submitted version.

## Conflict of Interest

The authors declare that the research was conducted in the absence of any commercial or financial relationships that could be construed as a potential conflict of interest.

## Abbreviations

D: Level difference isolation index
DnT: Standardized level difference isolation index
HVAC: Heating, ventilation, and air conditioning
L_in_: Noise level inside the incubator
L_in_63_, L_in_125_, L_in_250_, L_in_500_, L_in_1000_, L_in_2000_, L_in_4000_: Noise level inside the incubator at the band frequencies of 63, 125, 250, 500, 1,000, 2,000, and 4,000 Hz, respectively
L_out_: Noise level outside the incubator
L_out_63_, L_out_125_, L_out_250_, L_out_500_, L_out_1000_, L_out_2000_, L_out_4000_: Noise level outside the incubator at the band frequencies of 63, 125, 250, 500, 1,000, 2,000, and 4,000 Hz, respectively
Nabs: Room with acoustic absorbent covering
NICU: Neonatal intensive care unit
Nreverb: Room with reverberant surfaces
R′: Apparent sound reduction index
RIR: Impulsive response of a room
RT: Reverberation time
RT_in_: Reverberation time inside the incubator
RT_in_63_, RT_in_125_, RT_in_250_, RT_in_500_, RT_in_1000_, RT_in_2000_, RT_in_4000_: Reverberation time inside the incubator at the band frequencies of 63, 125, 250, 500, 1,000, 2,000, and 4,000 Hz, respectively
RT _in_Nreverb_: Reverberation time inside the incubator measured in the room with reverberant surfaces
RT_out_: Reverberation time outside the incubator
RT_out_63_, RT_out_125_, RT_out_250_, RT_out_500_, RT_out_1000_, RT_out_2000_, RT_out_4000_: Reverberation time outside the incubator at the band frequencies of 63, 125, 250, 500, 1,000, 2,000, and 4,000 Hz, respectively
SPL: Sound Pressure level
RT_out_Nreverb_: Reverberation time outside the incubator measured in the room with reverberant surfaces
T_0_: Reference reverberation time of the standardized level difference equation defined by the ISO16283-1
T: Reverberation time of the receiver room within the standardized level difference equation
T20: Time in which the sound pressure level decays 20 dB multiplied by 3
T30: Time in which the sound pressure level decays 30 dB multiplied by 2.

## References

[1] Williams AL Drongelen W Van Lasky RE. Noise in contemporary neonatal intensive care. J Acoust Soc Am. (2007) 2681:2681–90. 10.1121/1.2717500 17550168

[2] Lasky RE Williams AL. Noise and light exposures for extremely low birth weight newborns during their stay in the neonatal intensive care unit. Pediatrics. (2009) 123:540–6. 10.1542/peds.2007-3418 19171620

[3] Santos J Santos J Carvalhais C Xavier A Silva MV Santos J. Assessment and characterization of sound pressure levels in Portuguese neonatal intensive care units. Arch Environ Occup Heal ISSN. (2018) 73:121–7. 10.1080/19338244.2017.1304883 28287931

[4] Cardoso SMS Kozlowski L de C de Lacerda ABM Marques JM Ribas A. Newborn physiological responses to noise in the neonatal unit. Braz J Otorhinolaryngol. (2015) 81:583–8. 10.1016/j.bjorl.2014.11.008 26480903 PMC9442682

[5] Kuhn P Zores C Pebayle T Hoeft A Langlet C Escande B. Infants born very preterm react to variations of the acoustic environment in their incubator from a minimum signal-to-noise ratio threshold of 5 to 10 dBA. Pediatr Res. (2012) 71:386–92. 10.1038/pr.2011.76 22391640

[6] Wharrad HJ Davis AC. Behavioural and autonomic responses to sound in pre-term and full-term babies. Br J Audiol. (1997) 31:315–29. 10.3109/03005364000000026 9373741

[7] Vranekovic G Hock E Isaac P Cordero L. Heart rate variability and cardiac response to an auditory stimulus. Biol Neonate. (1974) 24:66–73. 10.1159/000240633 4830462

[8] Stanley N Graven M. Sound and the developing infant in the NICU: conclusions and recommendations for care. J Perinatol. (2000) 20:88–93. 10.1038/sj.jp.7200444 11190706

[9] American Academy of Pediatrics. Committee on Environmental Health. Noise: a hazard for the fetus and newborn. Pediatrics. (1997) 100:724–7. 10.1542/peds.100.4.724 9836852

[10] Environmental Protection Agency Office of Noise Abatement and Control. Information on Levels of Environmental Noise Requisite to Protect Public Health and Welfare With an Adequate Margin of Safety. Washington, DC: Government Printing Office (1974).

[11] Knutson AJ Acceptable noise levels for neonates in the neonatal intensive care unit. In: Independent Studies and Capstone Projects Program in Audiology and Communication Sciences. Washington, DC: University School of Medicine (2012).

[12] Darcy A Hancock L Ware E A descriptive study of noise in the neonatal intensive care unit ambient levels and perceptions of contributing factors. Adv Neonatal Care. (2008) 8:165–75. 10.1097/01.ANC.0000324341.24841.6e 18535422

[13] AggarwaL D Chawla S Rao D Basirico J Turn down the volume: a study of excessive sound levels in the neonatal intensive care unit. Pediatrics. (2019) 144:693 Available online at: https://pediatrics.aappublications.org/content/144/2_MeetingAbstract/693

[14] Smith SW Ortmann AJ Clark WW. Noise in the neonatal intensive care unit: a new approach to examining acoustic events. Noise Heal. (2018) 95:121–30. Available online at: https://www.ncbi.nlm.nih.gov/pmc/articles/PMC6122266/30136672 10.4103/nah.NAH_53_17 PMC6122266

[15] Carvalhais C Santos J Vieira M Xavier A. Is there sufficient training of health care staff on noise reduction in neonatal intensive care units? A Pilot Study From Neonoise. J Toxicol Environ Heal Part A Curr Issues. (2015) 78:897–903. 10.1080/15287394.2015.1051204 26167755

[16] Liu WF. The impact of a noise reduction quality improvement project upon sound levels in the open-unit-design neonatal intensive care unit. J Perinatol. (2010) 30:489–96. 10.1038/jp.2009.188 20010612

[17] Wang D Aubertin C Barrowman N Moreau K Dunn S Harrold J. Reduction of noise in the neonatal intensive care unit using sound-activated noise meters. Arch Dis Child Fetal Neonatal Ed. (2014) 99:F515–6. 10.1136/archdischild-2014-306490 25154983

[18] Coston AD Aune C Reducing noise in the neonatal intensive care unit. Pediatrics. (2019) 144:154 Available online at: https://pediatrics.aappublications.org/content/144/2_MeetingAbstract/154

[19] Spence J Decasper J Prenatal experience with sounds influence neonatal perception of maternal voice samples. Inf Behav Dev. (1987) 10:133–42. 10.1016/0163-6383(87)90028-2

[20] Abrams RM Gerhardt KJ. The acoustic environemnt and physilogical responses of the fetus. J Perinatol. (2000) 20:30–5. 10.1038/sj.jp.7200445 11190698

[21] Zahr LK Balian S. Responses of premature infants to routine nursing interventions and noise in the NICU. Nurs Res. (1995) 44:179–85. 10.1097/00006199-199505000-00009 7761295

[22] Peng N Bachman J Jenkins R Chen C Chang Y. Relationships between environmental stressors and stress biobehavioral responses of preterm infants in NICU. Adv Neonatal Care. (2013) 13:2–10. 10.1097/ANC.0000000000000023 24042180

[23] Field TM Dempsey JR Hatch J Ting G Clifton RK Cardiac and behavioral responses to repeated tactile and auditory stimulation by preterm and term neonates. Dev Psychol. (1979) 15:406–16. 10.1037/0012-1649.15.4.406

[24] Hassanein SMA El Raggal NM Shalaby AA. Neonatal nursery noise: practice-based learning and improvement. J Matern Neonatal Med. (2013) 26:392–5. 10.3109/14767058.2012.733759 23190305

[25] Vohr BR Screening the Newborn for hearing loss. Wolter Kluwer; UpToDate (2020)

[26] Lahav A Skoe E. An acoustic gap between the NICU and womb: a potential risk for compromised neuroplasticity of the auditory system in preterm infants. Front Neurosci. (2014) 8:381. 10.3389/fnins.2014.00381 25538543 PMC4256984

[27] Lejeune F Parra J Berne-audéoud F Marcus L. Sound interferes with the early tactile manual abilities of preterm infants. Nat Publ Gr. (2016) 6:1–8. 10.1038/srep23329 26987399 PMC4796902

[28] Lai TT Bearer CF. Iatrogenic environmental hazards in the neonatal intensive care unit. Clin Perinatol. (2008) 35:163–81. 10.1016/j.clp.2007.11.003 18280881 PMC3191461

[29] Long JG Lucey JF Philip AGS. Noise and hypoxemia in the intensive care nursery. Pediatrics. (1980) 65:143–5. 10.1542/peds.65.2.203 7355011

[30] Wachman EM Lahav A. The effects of noise on preterm infants in the NICU. Arch Dis Child Fetal Neonatal Ed. (2011) 96:F305–9. 10.1136/adc.2009.182014 20547580

[31] Williams AL Sanderson M Ph D Lai D Ph D. Intensive care noise and mean arterial blood pressure in extremely low-birth-weight neonates. Am J Perinatol. (2008). 26:323–9. 10.1055/s-0028-1104741 19085678

[32] Gray L Philbin MK Measuring sound in Hospital nurseries. J Perinatol. (2001) 20:99–103. 10.1038/sj.jp.7200440 11190688

[33] Hernandez Molina R Fernández Zacarías F Puyana Romero V Rodríguez Montaño VM Beira Jiménez JL Cueto Ancela JL Análisis del ambiente sonoro e una unidad de cuidados intersivos de neonatología. In: *XI Congreso Iberoamericano de Acústica; X Congreso Ibérico de Acúsica; 49*^*o*^ *Congreso Español de Acústica -TECNIACUSTICA'18* Cádiz (2018).

[34] Gareth PJ Lukashkina VA Russell IJ Lukashkin1 AN. The vestibular system mediates sensation of low-frequency sounds in mice. J Assoc Res Otolaryngol. (2010) 732:725–32. 10.1007/s10162-010-0230-7 20821033 PMC2975890

[35] Ohgami N Oshino R Ninomiya H Li X Kato M. Risk assessment of neonatal exposure to low frequency noise based on balance in mice. Front Behav Neurosci. (2017) 11:1–7. 10.3389/fnbeh.2017.00030 28275341 PMC5319995

[36] Fernández Zacarías F Beira Jiménez JL Bustillo Velázquez-Gaztelu PJ Hernández Molina R Lubián López S. Noise level in neonatal incubators: A comparative study of three models. Int J Pediatr Otorhinolaryngol. (2018) 107:150–4. 10.1016/j.ijporl.2018.02.013 29501298

[37] International Organization for Standardization Insulation, ISO 16283-1:2014. Acoustics — Field Measurement of Sound Insulation in Buildings and of Building Elements — Part 1: Airborne Sound. (2014) Available online at: https://www.iso.org/standard/55997.html (accessed September 07, 2020).

[38] Núñez-Solano D Puyana-Romero V Ordoñez-Andrade C Bravo-Monayo L Garzón-Pico C Impulse response simulation of a small room and in situ measurements validation. In: 147th Audio Engineering Society Convention. New York, NY (2019). p. 1–7.

[39] Puyana-Romero V Núñez-Solano D Hernández R Fernández-Zacarías F Beira-Jiménez JL Garzón C Reverberation time measurements of a neonatal incubator. Appl Acoust. (2020) 167:107374 10.1016/j.apacoust.2020.107374

[40] Kleiner M Tichy J *Acoustics of Small Rooms* (CRC Press. Taylor & Francis Group) (2014). p. 1–453.

[41] Berzborn M Bomhardt R Klein J Richter J-G Vorländer M The ITA-Toolbox: an open source MATLAB toolbox for acoustic measurements and signal processing. In: 43th Annu Ger Congr Acoust (2017). p. 222–5. Available online at: http://www.ita-toolbox.org/publications/ITA-Toolbox_paper2017.pdf (accessed September 07, 2020).

[42] Nachar N The Mann - Whitney U: a test for assessing whether two independent samples come from the same distribution. Tutor Quant Methods Psychol. (2008) 4:13–20. 10.20982/tqmp.04.1.p013

[43] Pinheiro EM Guinsburg R Nabuco MA de A Kakehashi TY. Noise at the neonatal intensive care unit and inside the interior of the incubator (in spanish). Rev Lat Am Enfermagem. (2011) 19:1214–21. 10.1590/S0104-11692011000500020 22030587

[44] Rodarte MDO Scochi CGS Leite AM Fujinaga CI Zamberlan NE Castral TC. O ruído gerado durante a manipulação das incubadoras: implicações para o cuidado de enfermagem. Rev Lat Am Enfermagem. (2005) 13:79–85. 10.1590/S0104-11692005000100013 15761584

[45] Mašovi D Member S Mete Ö Analysis of reverberation time field measurement results in building acoustics. Telfor J. (2013) 5:145–50. Available online at: http://journal.telfor.rs/Published/Vol5No2/Vol5No2.aspx (accessed September 07, 2020).

[46] Tamura H Ohgami N Yajima I Iida M Ohgami K Fujii N. Chronic exposure to low frequency noise at moderate levels causes impaired balance in mice. PLoS ONE. (2012) 7:1–7. 10.1371/journal.pone.0039807 22768129 PMC3387207

